# Individual Placement and Support for persons with mental disorders and disability pension: Randomized controlled trial and follow up

**DOI:** 10.1192/j.eurpsy.2024.178

**Published:** 2024-08-27

**Authors:** W. Kawohl

**Affiliations:** ^1^Clienia Schlössli AG, Oetwil am See; ^2^Psychiatry, Psychotherapy and Psychosomatics, University of Zurich, Zurich, Switzerland; ^3^University of Nicosia Medical School, Nicosia, Cyprus

## Abstract

**Introduction:**

Individual Placement and Support (IPS) is a supported employment method used for the vocational inclusion of individuals with mental disorders. There is vast evidence that IPS is effective for finding jobs. However, evidence concerning the applicability of IPS for persons with mental disorders receiving a disability pension and concerning the sustainability of IPS is scarce.

**Objectives:**

The aim of the studies included in this report was to a) control for the applicability of IPS for persons receiving a disability pension and b) to gain insight in the sustainability of IPS in this context.

**Methods:**

A randomized controlled trial with 250 participants was conducted. The participants in the intervention group received job coaching according to the IPS standard. Members of the control group received no organized support but were allowed to seek assistance on their own. The initial phase of the study lasted 24 month. Job coaching was delivered only in the first phase of the study and discontinued after 24 month. A follow up was performed six years after the start of the study to clarify the further course (number of employment relationships, degree of employment, duration of employment, salary).

**Results:**

In the first phase of the study, the overall dropout rate was 32%. 114 participants (46% of the original number of participants) took part in the follow-up survey. The intervention was superior to the control condition in the first phase. There were no significant differences between the groups in terms of number of employment relationships, degree of employment, length of employment, and salary in the follow up.

**Conclusions:**

The effect of a clear superiority of the IPS intervention with regard to the number of employment relationships, which was measured during the originally planned duration of the study, was only slightly detectable six years after the start of the study and up to four years after the end of the intervention and was no longer statistically significant. This result underlines the importance of continuing job coaching for an unlimited period of time, as called for in the IPS concept, in order to perpetuate the positive effects such as finding and maintaining a job in the primary labor market.

**Disclosure of Interest:**

None Declared

